# Evaluation of RT-qPCR and Loop-Mediated Isothermal Amplification (LAMP) Assays for the Detection of SARS-CoV-2 in Argentina

**DOI:** 10.3390/genes12050659

**Published:** 2021-04-28

**Authors:** María Dolores Fellner, Romina Bonaventura, Jorge Basiletti, Martín Avaro, Estefanía Benedetti, Ana Campos, María Elena Dattero, Mara Russo, Sara Vladmirsky, Viviana Molina, Lucía Irazu, Marcelo A. Rodriguez, Andrea Pontoriero, Daniel M. Cisterna, Elsa G. Baumeister

**Affiliations:** 1 Servicio de Virus Oncogénicos, Departamento de Virología, Instituto Nacional de Enfermedades Infecciosas—ANLIS Dr. Carlos G. Malbrán, Ciudad Autónoma de Buenos Aires 1282AFF, Argentina; mdfellner@anlis.gob.ar (M.D.F.); jabasiletti@anlis.gob.ar (J.B.); 2 Servicio de Neurovirosis, Departamento de Virología, Instituto Nacional de Enfermedades Infecciosas—ANLIS Dr. Carlos G. Malbrán, Ciudad Autónoma de Buenos Aires 1282AFF, Argentina; rbonaventura@anlis.gob.ar; 3 Servicio de Virosis Respiratorias, Departamento de Virología, Instituto Nacional de Enfermedades Infecciosas—ANLIS Dr. Carlos G. Malbrán, Ciudad Autónoma de Buenos Aires 1282AFF, Argentina; mavaro@anlis.gob.ar (M.A.); ebenedetti@anlis.gob.ar (E.B.); acampos@anlis.gob.ar (A.C.); medattero@anlis.gob.ar (M.E.D.); mrusso@anlis.gob.ar (M.R.); aponto@anlis.gob.ar (A.P.); ebaumeister@anlis.gob.ar (E.G.B.); 4 Servicio de Hepatitis Virales, Departamento de Virología, Instituto Nacional de Enfermedades Infecciosas—ANLIS Dr. Carlos G. Malbrán, Ciudad Autónoma de Buenos Aires 1282AFF, Argentina; svladimirsky@anlis.gob.ar; 5 Instituto Nacional de Enfermedades Infecciosas—ANLIS Dr. Carlos G. Malbrán, Ciudad Autónoma de Buenos Aires 1282AFF, Argentina; vmolina@anlis.gob.ar; 6 Unidad de Evaluación de Métodos de Diagnóstico y Estadística Aplicada, Instituto Nacional de Enfermedades Infecciosas—ANLIS Dr. Carlos G. Malbrán, Ciudad Autónoma de Buenos Aires 1282AFF, Argentina; lirazu@anlis.gob.ar (L.I.); marcerodriguez2002@gmail.com (M.A.R.)

**Keywords:** SARS-CoV-2, RT-qPCR, RT-LAMP

## Abstract

Our aim was to evaluate the analytical and clinical performance of the SARS-CoV-2 molecular detection kits used in Argentina. Nine real-time reverse-transcription polymerase chain reaction (RT-qPCR) and three reverse-transcription loop-mediated isothermal amplification (RT-LAMP) assays were evaluated using the World Health Organization (WHO) recommended test as reference method. A secondary standard calibrated for the E, N and RdRp genes against the Pan American Health Organization—World Health Organization—International Standard was used to calculate the limit of detection (LoD). A panel of artificial clinical samples, 32 positive and 30 negative for SARS-CoV-2, were analyzed to estimate the kappa concordance (κ) and the diagnostic performance. Differences among the LoD values for the target genes amplified by each kit were >1 log copies/reaction. The κ for the RT-qPCR kits was greater than 0.9, whereas that for the RT-LAMP assays ranged from 0.75 to 0.93. The clinical performance of RT-qPCR kits showed 100% specificity and high sensitivity, although with variations according to the gene analyzed. The E and N genes provided greater clinical sensitivity, whereas the RdRp gene increased the clinical specificity. The RT-LAMP assays revealed a variable diagnostic performance. The information provided can be useful to choose the most appropriate diagnostic test and may contribute to the establishment of a consensus in the diagnosis of SARS-CoV-2 in Argentina and the region.

## 1. Introduction

Coronavirus disease 2019 (COVID-19) is caused by the novel coronavirus SARS-CoV-2, the only *Sarbecovirus* currently circulating in humans [1]. The laboratory testing for COVID-19 confirmation recommended by the World Health Organization (WHO) is the detection of SARS-CoV-2 RNA by nucleic acid amplification testing, such as real-time reverse-transcription polymerase chain reaction (RT-qPCR) [2]. Several molecular assays are available within the WHO Emergency Use List (EUL) (https://www.who.int/diagnostics_laboratory/EUL/en/ accessed on: 12 February 2021). These assays are either further independently evaluated by the Foundation for Innovative New Diagnostics (FIND), a WHO Collaborating Center (https://www.finddx.org/covid-19/sarscov2-eval-molecular/molecular-eval-results/ accessed on: 12 February 2021), or approved for commercialization by National Regulatory Authorities. Optimal COVID-19 diagnosis consists in detecting at least two independent targets in the SARS-CoV-2 genome. However, in areas with widespread transmission, one single discriminatory target might be acceptable. Additionally, other amplification methods, such as isothermal nucleic acid amplification technologies (e.g., reverse transcription loop-mediated isothermal amplification, RT-LAMP), have emerged as useful alternatives in the context of the current COVID-19 pandemic [3,4]. However, validation of the analytical and clinical performance of these assays is encouraged to increase access to reliable SARS-CoV-2 testing.

In Argentina, the Service of Respiratory Viral Diseases of the National Reference Laboratory INEI-ANLIS “Dr. Carlos G. Malbrán” promptly adopted the reference technique developed by the Charité Institute of Virology (Germany) and recommended by WHO, which detects the E and RdRp genes and allowed diagnosing the first cases of COVID-19 [5]. However, the urgent need for diagnosis caused by the pandemic introduced a wide range of commercial tests, many of which were previously unknown in the local market. In addition, the national government promoted the development of new tests by the public and private scientific system.

Facing the health emergency, Argentinean laboratories began to make the diagnosis of COVID-19 by using the tests at their disposal. Although the National Administration of Drugs, Foods and Medical Technology (ANMAT) published a list of authorized tests in the context of the health emergency (https://www.argentina.gob.ar/anmat/regulados/productos-medicos/reactivos-covid-19#1 accessed on: 12 February 2021), in many cases their performance has not yet been well established. Thus, the aim of our study was to evaluate and compare the analytical and clinical performance of nine different RT-qPCR assays and three RT-LAMP assays of SARS-CoV-2 used in Argentina.

## 2. Materials and Methods

### 2.1. Study Design

A comparison-of-methods study was conducted, in which commercial SARS-CoV-2 RNA detection assays were compared against the WHO recommended RT-qPCR test, as the reference method.

### 2.2. Preparation and Calibration of a Secondary Standard

SARS-CoV-2 RNA was extracted from a viral isolate obtained in the Vero cell line (ATCC, CCL-81) from a clinical sample using the QIAamp Viral RNA mini kit (QIAGEN, Hilden, Germany) (CoV-19/Argentina/C121/2020/EPI_ISL_420600/2020-03-07). To calibrate the secondary standard, the E, RdRp and N genes, from both the PAHO-WHO International Standard (SARS-like Wuhan ivRNA E, RdRp and N Genes; 1 × 10^8^ copies/µL) and the SARS-CoV-2 RNA extracted were quantified in parallel from tenfold serial dilutions (10^−1^–10^−6^), each replicated six times, for two days, using the WHO recommended RT-PCR test. The potency of each gene was estimated by applying the parallel-line model (http://www.anmat.gov.ar/webanmat/fna/pfds/Farmacopea_Argentina_2013_Ed.7.pdf, accessed on 12 February 2021). The product obtained was called “SARS-CoV-2 Secondary Standard, RNA 002/20 batch, E, RdRp and N genes” (SARS-CoV-2 SStd) and was aliquoted and stored at −80 °C.

### 2.3. Panel of Positive and Negative SARS-CoV-2 Contrived Samples

Thirty-two pools of positive SARS-CoV-2 contrived samples were prepared by spiking different quantities of the SARS-CoV-2 SStd in a pool of RNAs extracted from clinical samples, which had been previously tested as negative for SARS-CoV-2 by applying the WHO recommended test. The contrived samples encompassed concentrations from 10^5^ to 5 copies/µL. Likewise, thirty pools of negative SARS-CoV-2 contrived samples were generated by mixing groups of RNAs extracted from clinical samples, previously tested as negative for SARS-CoV-2 by the WHO recommended test. To standardize the evaluation of the RT-PCR assays, 5 µL of each of the artificial samples were aliquoted in real-time PCR plates, which were kept at −80 °C until use. In addition, fresh clinical samples, twenty-seven positive and thirty negative for SARS-CoV-2 according to the results of the WHO recommended assay, were selected to analyze the performance of the iAMP COVID-19 Detection Kit (ATILA Biosystems, Mountain View, CA, USA) since it includes an RNA extraction step using lysis buffer. The positive samples were selected taking into account that their Ct values were similar to those observed in the panel of contrived samples.

### 2.4. SARS-CoV-2 RNA Detection Kits

The WHO recommended method was used as the reference technique to detect the E, N and RdRp genes, as previously described [5]. Furthermore, the following nine RT-qPCR commercial kits were assessed: RealStarSARS-CoV-2 RT-PCR Kit 1.0 (Altona Diagnostics GmbH, Hamburg, Germany), Detection kit for 2019 Novel coronavirus (2019-nCoV) RNA (Da An Gene Ltd., Guangzhou, China), AccuPower SARS-CoV-2 Realtime RT PCR Kit (Bioneer Corporation, Daejeon, Korea), VIASURE SARS CoV-2 Real time PCR Detection Kit (CerTest Biotec SL, Zaragoza, Spain), GeneFinder COVID-19 Plus RealAmp kit (Osang Healthcare Co., Ltd., Gyeonggi-do, Korea), ARGENE SARS-COV-2 R-GENE^®^ (Biomerieux, Marcy Letoile, France), CoronARDx SARS-CoV-2 RT-PCR Kit (Argenomics ZevBiotech, Buenos Aires, Argentina), and WGene SARS-CoV-2 RT detection kit (Wiener lab, Rosario, Argentina). Additionally, the following three LAMP assays were evaluated: iAMP COVID-19 Detection Kit (ATILA Biosystems), Covid-19 NEOKIT Tecnoami, and ELA CHEMSTRIP COVID-19 (Chemtest Argentina S.A., Buenos Aires, Argentina). Each kit was used to detect SARS-CoV-2 RNA as recommended by the manufacturers. An overview of the kits analyzed is presented in Table 1.

### 2.5. Comparison of Methods and Statistical Analyses

Replicates of tenfold plus twofold serial dilutions of the SARS-CoV-2 SStd were used to estimate the 95% limit of detection (LoD_95%_) and corresponding confidence interval of each kit by applying the POD-LOD v9 software (https://www.wiwiss.fu-berlin.de/fachbereich/vwl/iso/ehemalige/wilrich/PODLOD_ver9.xls accessed on: 4 January 2021). The calibrated SARS-CoV-2 SStd allowed estimating the LoD_95%_ expressed as copies per reaction or logarithm units of copies per reaction [6].

The panel of contrived samples was analyzed with each kit to determine the concordance with the recommended WHO test and the clinical sensitivity and specificity, using the Analyse-it v 30.2 software (https://analyse-it.com/ accessed on: 4 January 2021). Cohen’s kappa values (κ) were calculated, with values categorized as follows: >0.90 = almost perfect, 0.90 to 0.80 = strong, 0.79 to 0.60 = moderate, 0.59 to 0.40 = weak, 0.39 to 0.21 =minimal, and 0.20 to 0 = none.

## 3. Results

### 3.1. Calibration of the Secondary Standard

**Figure 1:**
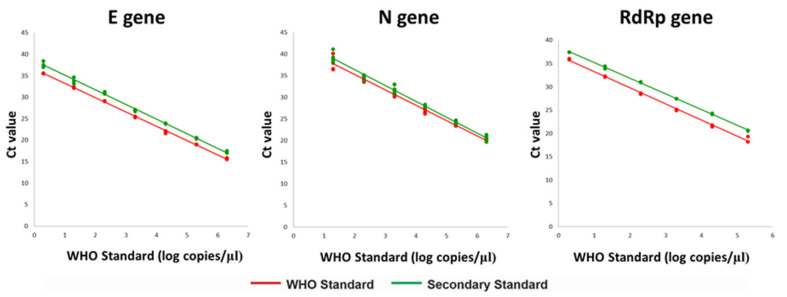
Calibration of the secondary standard against the WHO Reference Standard. The Ct values obtained in the parallel amplification of the E, N, and RdRp genes from different concentrations of the SARS-CoV-2-SStd (green line) and the WHO Reference Standard (red line) are graphed.

The Ct values obtained in the detection of the E, N and RdRp genes when examining parallel serial dilutions of the SARS-CoV-2 SStd and the PAHO-WHO Standard are shown in Figure 1. The parallel-line model yielded concentrations of 3 × 10^7^ copies/μL, 6 × 10^7^ copies/μL, and 2 × 10^7^ copies/μL for the E, N and RdRp genes respectively. It should be noted that the PAHO-WHO Standard was not calibrated for the S gene. 

### 3.2. Differences in the Limit of Detection of Commercial Kits

Analysis of the LoD for all kits allowed us to confirm the information of the manufacturers, except for the ARGENE (observed LoD 27.0–126.0 copies per reaction), the WGene SARS-CoV-2 RT detection kit of Wiener lab (observed LoD 23.0–102.0 copies per reaction) and the NEOKIT (observed LoD 115.0–367.0 copies per reaction), which showed a LoD value higher than that declared. These differences could be due to the fact that the manufacturers of the ARGENE and WGene kits determined the analytical sensitivity of their assays from dilutions of a viral culture or a commercial control (Vircell, Granada, Spain), respectively, both different from the SARS-CoV-2 SStd. In the case of the Altona Diagnostics test, which can detect the E and the S genes, the LoD was estimated only for the E gene. The WGene SARS-CoV-2 RT detection kit, the iAMP COVID-19 Detection Kit (ATILA Biosystems), and the Covid-19 NEOKIT-Tecnoami gave a single signal for SARS-CoV-2 positive result, thus not allowing us to discriminate which gene was amplified.

**Figure 2:**
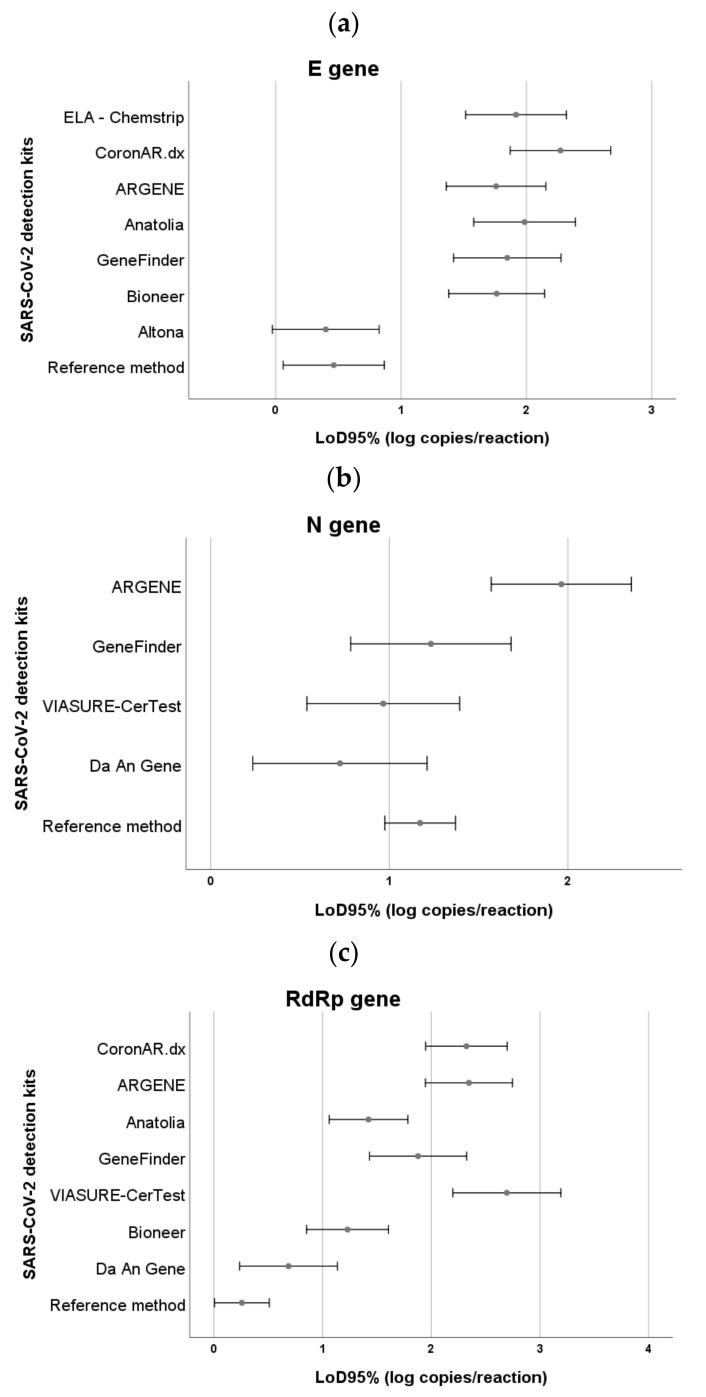
SARS-CoV-2 LoD_95%_ detection kits according to the target genes amplified. (**a**) E gene, (**b**) N gene, (**c**) RdRp gene. The lower and upper confidence intervals of the LoD_95%_ corresponding to each of the target genes amplified by the detection kits are shown. In the case of the assays giving a single signal without discriminating each target gene, the LoD_95%_ values observed were as follows: WGene SARS-CoV-2 RT detection kit: 23–102 copies per reaction (1.36–2.01 log copies/reaction), iAMP COVID-19 Detection Kit (ATILA Biosystems): 30–60 copies per reaction (1.48–1.78 log copies/reaction) and Covid-19-NEOKIT-Tecnoami: 115–367 copies per reaction (2.06–2.56 log copies/reaction).

Additionally, differences greater than one logarithmic unit were observed between the estimated LoD values for the different target genes amplified by each kit (Figure 2). The Altona and Da An Gene kits showed, respectively, the lowest LoD for the E gene (LoD 1.1–6.0 copies per reaction) and RdRpgene (LoD 2.0–12.0 copies per reaction), resulting similar to the method recommended by WHO (LoD-E gene: 1.3–7.0 copies per reaction; LoD-RdRp gene: 1.1–3.0 copies per reaction). The Da An Gene (LoD 2.0–14.0 copies per reaction), VIASURE CerTest (LoD 4.0–22.0 copies per reaction) and GeneFinder (LoD 7.0–42.0 copies per reaction) assays showed the lowest LoD for the N gene, equivalent to the reference test (LoD_N-gene_: 10.0–22.0 copies per reaction).

### 3.3. Variations in the Clinical Performance of the Commercial Kits

**Figure 3:**
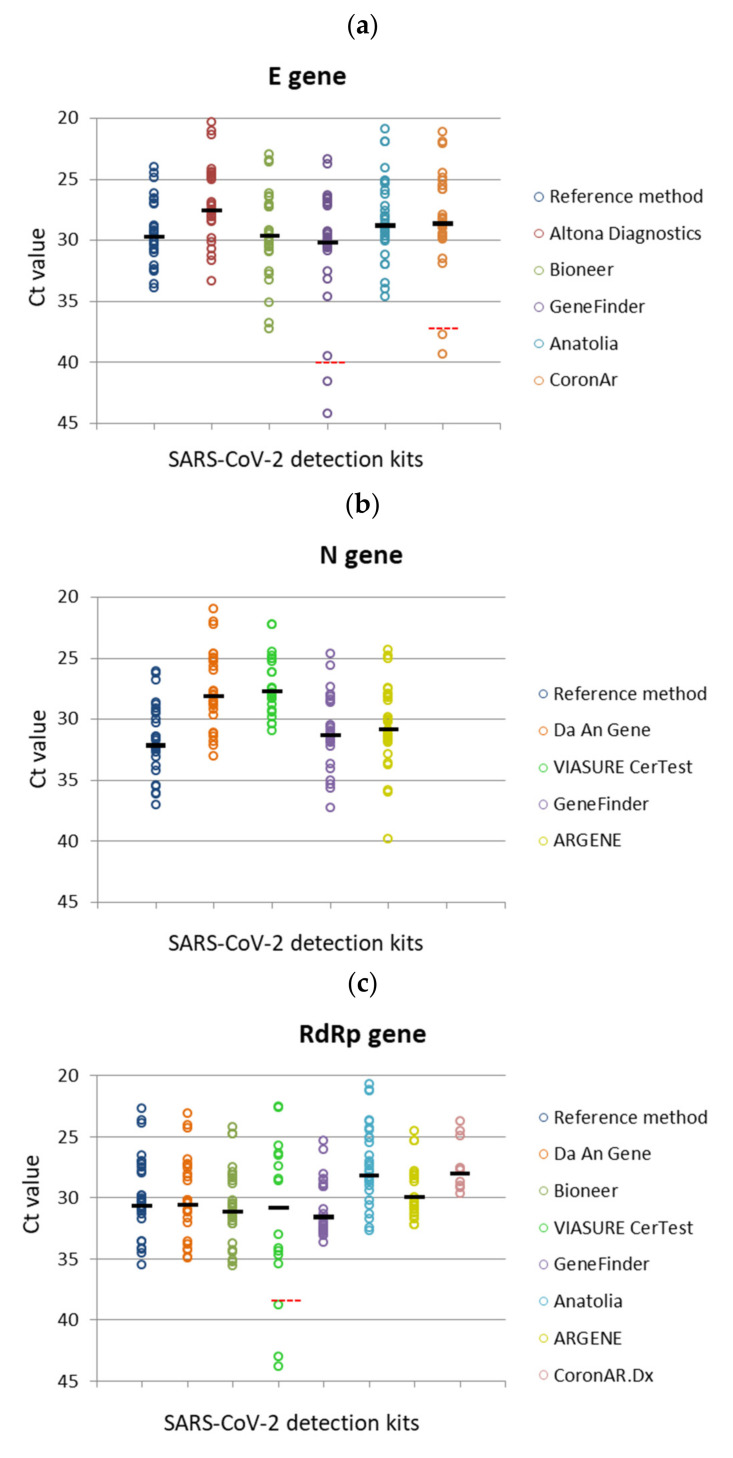
Distribution of the Ct values for the genesdetected using the RT-qPCR detection kits. (**a**) E gene, (**b**) N gene, (**c**) RdRp gene. The Ct values observed for the E, N and RdRp target genes during the amplification of the panel of positive samples with each of the RT-qPCR kitsare depicted. The circles correspond to the Ct values reached by each of the target genes amplified with the kits analyzed. The ratio above the circles indicates the rate of positive results regarding the total number of samples of the panel.

The study of the positive contrived samples showed discrepancies according to the gene analyzed and yielded variations in the Ct values obtained with each RT-qPCR kit (Figure 3). The E and N genes were detected in most of the samples, with the exception of 2/32 (E gene) and 1/32 (N gene), corresponding to the specimens with the lowest viral loads, whereas the RdRp gene presented wide variations in its detection rate, ranging from 15/32 to 32/32 of the positive samples. Moreover, a similar distribution pattern of Ct values was observed for each of the three genes evaluated, although shifting to higher or lower values regarding the reference method. This displacement fluctuated between four Ct values lower (N gene, VIASURE CerTest) to around two Ct values higher (RdRp gene, GeneFinder) than those obtained with the assay of the Charité Institute.

The concordance with the reference method was greater than 0.9 for the RT-qPCR kits, whereas it ranged from 0.75 to 0.93 for the RT-LAMP assays (Table 2). The clinical performance of the RT-qPCR kits showed a specificity of 100% in almost all cases and high sensitivity, although with variations according with the gene analyzed. The E and N genes showed greater clinical sensitivity: the VIASURE CerTest (Ngene: 100%), GeneFinder (Egene: 96.8%; N gene: 100%), ARGENE (Ngene: 100%) and CoronAR (E gene: 93.6%) kits, whereas the RdRp gene in almost all kits or the S gene in the case of the Altona kit contributed to increasing the clinical specificity. On the other hand, the RT-LAMP assays revealed a variable diagnostic performance. The CHEMSTRIP Kit showed high clinical sensitivity (100%), whereas both the iAMP COVID-19 Detection Kit and the NEOKIT exhibited high clinical specificity (100%).

## 4. Discussion

In this study, we evaluated the analytical sensitivity and clinical performance of twelve molecular assays (nine RTqPCR and three RT-LAMP assays) for the detection of SARS-CoV-2 available in Argentina by comparing them with the WHO recommended test. To this end, we developed a secondary SARS-CoV-2 RNA standard from a clinical isolate and calibrated it against a WHO reference standard [7].

The detection of SARS-CoV-2 infection is based on the amplification of genome sequences located in the E, N, RdRp and S genes. Each manufacturer indicates the interpretation of a positive test depending on which viral gene or genes are detected. Therefore, the analytical sensitivity for the detection of the different viral targets will affect the clinical performance of an assay.

In agreement with what is described by Corman et al., the analysis of the reference method of the Charité Institute performed in the present study showed a barely lower LoD for the E and RdRp genes than for the N gene. However, among the different targets detected by the commercial kits evaluated in this report, the N gene presented the lowest LoD except with the ARGENE assay. On the other hand, the RdRp gene/ORF1abshowed the lowest analytical sensitivity with most tests, except with Bioneer and Anatolia; besides, in agreement with the data of other authors, a wide variation among kits was also observed [8,9,10].Interestingly, when analyzing all the target genes together, the Altona and Da An Gene assays reached an analytical sensitivity equal to or greater than that reported for the detection of the E gene using the reference method, while the Bioneer, GeneFinder, VIASURE CerTest and Anatolia assays achieved a slightly higher LoD.

LAMP tests have been proposed as an interesting option for the diagnosis of SARS-CoV-2 infection because they are rapid, sensitive and effective visual nucleic acid amplification methods [3,11,12]. Several reports have described the development of this type of assays for SARS-CoV-2 detection [13,14,15], and WHO encourages the analysis of their analytical and clinical performance, so as to demonstrate their potential operational utility and sharing of data [16]. In agreement with previous reports, all the RT-LAMP tests analyzed in our study showed a lower analytical sensitivity than that observed with the reference assay [17,18,19]. Several RT-LAMP tests which amplify either an individual or a combination of different viral targets have shown a LoD ranging around 20–200 copies per reaction [17,20,21,22]. These values are similar to those observed in our study and represent a 10–100-fold higher amount [16] than the lowest viral quantity detected by the reference method. Unlike RT-qPCR tests, the analytical sensitivity of RT-LAMP assays does not seem to be mainly related to the target genes, since up to tenfold higher differences in the LoD of the assay have been reported when detecting the same viral regions [20,21] or even a different number of targets [22]. Likewise, in the present report, we observed differences in the LoD among the LAMP tests analyzed, regardless of the target gene/s that each test amplifies.

On the other hand, the analysis of the panel of clinical samples showed that almost all RT-qPCR kits evaluated presented a very good concordance with the reference method and high diagnostic performance. The only exception corresponded to the VIASURE CerTest kit, due to the lack of detection of the ORF1ab gene in the positive samples with lower viral load, which is in agreement with its high LoD. However, when considering only the N gene, both the concordance with the WHO recommended test and the diagnostic performance were optimal. Regarding the clinical specificity, all the RT-qPCR tests evaluated achieved the highest performance following the manufacturer’s interpretation. Furthermore, the clinical sensitivity presented more variation, although it remained above 90% in all cases, except in the case of the VIASURE CerTest, as mentioned. When considering individual genes, the E and N genes showed high sensitivity, whereas the S gene (Altona Kit) and the RdRp/ORF1ab gene (most kits) showed high specificity, in agreement with other reports [23,24,25]. RT-LAMP tests showed a variable diagnostic performance, lower than that of the RT-qPCR assays. Some, such as the ELA CHEMSTRIP, demonstrated high clinical sensitivity, whereas others, such as the ATILA and NEOKIT tests, showed high clinical specificity. These data are in accordance with previous studies reporting lower diagnostic ability with respect to that of RT-qPCR methods for the detection of SARS-CoV-2 [26,27,28], although some previous reports have described analytical sensitivities similar to those of RT-PCR assays [29,30]. In this regard, most LAMP studies have shown that the diagnostic specificity is of less concern than the sensitivity [27]. Moreover, the use of crude patient samples has demonstrated lower diagnostic sensitivity than that of purified RNA, although it allows the procedure to be faster and easier [11]. The ATILA assay stands out because it includes a previous RNA extraction step, which reduces the procedure time. It should be noted that a version of NEOKIT (Neokit Plus), which includes a viral RNA isolation step that reduces the process time, has been recently launched, but was not analyzed in this study.

The combined LoD of both RT-qPCR and RT-LAMP assays shows that RT-qPCRs perform better than RT-LAMPs. However, RT-LAMPs have the advantage of being faster and requiring more accessible and less sophisticated equipment. Consequently, we recommend their use in areas of high viral circulation, although special consideration must be taken regarding the working areas in the laboratory since the LAMP technology is very susceptible to cross contamination.

Another important issue to consider in molecular tests for diagnostic purposes is the inclusion of an internal control, which allows detecting the presence of reaction inhibitors and analyzing the quality and quantity of the clinical sample [31,32]. It should be noted that an endogenous control is always preferable since it may also become a control of the validity of sampling, as well as the absence of the aforementioned reaction inhibitors. All RT-PCR methodologies evaluated in the present study include an internal control (endogenous or exogenous) either in a multiplex (Altona, Da An Gene, GeneFinder, VIASURE CerTest, Anatolia, ARGENE, and Wiener lab) or single (CoronAr) reaction format. Regarding LAMP assays, the ATILA test is a real-time fluorescent RT-isothermal assay, which includes a multiplex reaction for detection of SARS-CoV-2 (N gene plus ORF1ab) and an internal control. While LAMP assays with colorimetric detection cannot be multiplexed and another reaction tube must be added, as is the case of the ELA-CHEMSTRIP kit, it should be noted that the internal control reaction of this kit failed in 30% of the samples tested. The NEOKIT kit does not contain an internal control.

Most molecular assays applied to the diagnosis of covid-19 were developed based on the reference sequence Wuhan-1 strain (NC_045512.2), which at the time of this study, was the only identified strain. Since the emergence of variants/mutants of SARS-CoV-2, the identification of changes in primer-binding or probe-binding sites has received much attention, as they may affect the performance of nucleic acid diagnostic assays. Some reports describe substitutions involving the ORF1ab or the N-gene in genetic variants circulating in Colombia [33] as well as mutations associated with a failure in the amplification of the E, N and S targets genes [34,35,36]. Likewise, Sampaio Osório et al. reported that at least one of the designed primers from assays shared by WHO [16] is now likely to be ineffective for detecting up to 14% of the virus variants circulating around the world [37]. The use of assays targeting different viral regions would decrease this risk. It should be pointed out, that a continuous monitoring of viral genetic variants is essential to allow a rapid response in case there’s a need for assay redesign.

One of the limitations of the present study was the use of artificial samples instead of clinical samples and the small number of samples evaluated. In contrast, one of the strengths of the study was the use of a panel of samples maintained under the same conditions, a fact that favored the comparability of the results and the development and application of a calibrated secondary standard.

## 5. Conclusions

We believe that the information provided can be useful to choose the most appropriate diagnostic test and can contribute to the establishment of a consensus in the diagnosis of SARS-CoV-2 in Argentina and in other countries of the region.

## Figures and Tables

**Table 1:** Overview of the evaluated SARS-CoV-2 detection kits available in Argentina.

Kit	Methodology	Regulation Status	Country of Origin	Internal Control	No. PCRs (Target Genes) ^1^	Equipment	Ct ^2^ Cut-Off
WHO protocol- Charité assay (https://www.who.int/docs/default-source/coronaviruse/protocol-v2-1) (accessed on: 1 February 2020)	RT-qPCR	LDT ^3^	Germany	An endogenous human gene detection was used in an independent reaction	Three single PCRs (E,N,RdRp)	ABI 7500	Not indicated. Any signal is considered positive
RealStar SARS-CoV-2 RT-PCR Kit 1.0 (Altona Diagnostics)	RT-qPCR	CE-IVD ^4^	Germany	Heterologous. Spiked into sample or PCR mix.	One multiplex PCR (E,S,IC ^5^)	ABI 7500	Not indicated. Any signal is considered positive
Detection kit for 2019Novel coronavirus (2019-nCoV) RNA-Da An Gene Ltd. (Sun-Yat University)	RT-qPCR	CE-IVD	China	Pseudovirus containing human endogenous internal standard	One multiplex PCR (N, ORF1ab, IC)	ABI 7500	≤40
AccuPower SARS-CoV-2 Realtime RT PCR Kit (Bioneer Corporation)	RT-qPCR	CE-IVD	Korea	Heterologous. Included in PCR mix	Two multiplex PCRs (PCR_1_: E, IC PCR_2_: RdRP, IC)	CFX96	≤38
VIASURE SARS CoV-2 Real time PCR Detection Kit (CerTest Biotec SL)	RT-qPCR	CE-IVD	Spain	Heterologous. Included in PCR mix	One multiplex PCR (N,ORF1ab, IC)	CFX96	<38
GeneFinder COVID-19 Plus Real*Amp* kit (Osang Healthcare Co., Ltd.)	RT-qPCR	CE-IVD	Korea	Endogenous human IC	One multiplex PCR (E, N, RdRP, IC)	ABI 7500	≤43
Bosphore Novel Coronavirus (2019-nCoV) detection kit v2 (Anatolia Geneworks)	RT-qPCR	CE-IVD	Turkey	Heterologous. Spiked into sample or PCR mix	One multiplex PCR (E, ORF1ab, IC)	ABI 7500	Not indicated
ARGENE SARS-COV-2 R-GENE^®^ (Biomerieux)	RT-qPCR	CE-IVD	France	Two types: IC spiked into sample and cellular control	Two multiplex PCRs (PCR_1_: N, RdRp, IC PCR_2_: E, IC, C_human_)	CFX96	Not indicated
CoronAR.dx (Argenomics- ZEV-Biotech)	RT-qPCR	IVD	Argentina	Synthetic DNA corresponding to the RPP30 gene	Three single PCRs (E, RdRP, IC)	ABI 7500	≤37 ≤ 40 ≤ 35
WGene SARS-CoV-2 RT detection kit (Wiener lab)	RT-qPCR	CE-IVD	Argentina	Endogenous IC corresponding to RNAsaP	One multiplex PCR (N + RdRp), IC	ABI 7500	
iAMP COVID-19 Detection Kit (ATILA Biosystems)	Real time IA ^6^	IVD	China	Includes an IC to validate the extraction procedure	One multiplex IA (N + ORF1ab), IC	ABI 7500	Any signal is considered positive
Covid-19 NEOKIT Tecnoami (NEOKIT SAS)	Colorimetric IA	IVD-ANMaT ^7^	Argentina	Not included	One multiplex IA (E + N+ORF1Aa + ORF1ab)	Thermal block	NA ^8^
ELA CHEMSTRIP Covid-19 (Chemtest Argentina SA)	IA plus immune- chromatographic detection	ANMaT	Argentina	Endogenous IC corresponding to RNAsaP	Two single IA (E, IC)	Thermal block	NA

^1^ SARS-CoV-2 targets: E gene, N gene, RdRp gene, ORF1ab: open reading frame 1ab; ^2^ cycle threshold (according to the manufacturer); ^3^ LDT: laboratory-developed test, ^4^ CE-IVD: European Conformity-In Vitro Diagnostics; ^5^ IC: internal control; ^6^ IA: isothermal amplification; ^7^ ANMaT: National Administration for Drugs, Food and Medical Technology (Argentina); ^8^ NA: not applicable.

**Table 2:** Clinical performance of the different methodologies used for the detection of SARS-CoV-2 in Argentina.

Kits	Target Genes ^1^	Kappa Index (CI 95%)	Clinical Sensitivity (%) (CI 95%)	Clinical Specificity (%) (CI 95%)
Altona Diagnostics	E	0.97 (0.85–1)	100 (89.3–100)	96.7 (0.79–0.98)
	S	1.00 (1.00–1.00)	100 (89.3–100)	100 (88.6–100)
	E + S	1.00 (1.00–1.00)	100 (89.3–100)	100(88.6–100)
DaAn Gene	N	1.00 (1.00–1.00)	100 (89.3–100)	100 (88.6–100)
	ORF1ab	1.00 (1.00–1.00)	100 (89.3–100)	100 (88.6–100)
	N + ORF1ab	1.00 (1.00–1.00)	100 (89.3–100)	100 (88.6–100)
Bioneer	E	1.00 (1.00–1.00)	100 (89.3–100)	100 (89.0–100)
	RdRp	1.00 (1.00–1.00)	100 (89.3–100)	100 (89.0–100)
	E + RdRp	1.00 (1.00–1.00)	100 (89.3–100)	100 (89.0–100)
VIASURE-CerTest	N	1.00 (0.85–1.00)	100 (89.0 –100)	100 (79.3– 98.2)
	ORF1ab	0.42 (0.24–0.60)	42 (26.4–59.2)	100 (89.0–100)
	N + ORF1ab	0.42 (0.24–0.60)	42 (26.4–59.2)	100 (89.0–100)
GeneFinder	E	0.97 (0.91–1.00)	96.8 (83.8 –99.4)	100 (89.0–100)
	N	1.00 (1.00–1.00)	100 (89.0–100)	100 (89.0–100)
	RdRp	0.81 (0.66–0.95)	80.6 (63.7–90.8)	100 (89.0–100)
	E + N + RdRp	1.00 (1.00–1.00)	100 (89.0–100)	100 (89.0–100)
Anatolia	E	1.00 (1.00–1.00)	100 (89.3–100)	100 (89.0–100)
	ORF1ab	1.00 (1.00–1.00)	100 (89.3–100)	100 (89.0–100)
	E + ORF1ab	1.00 (1.00–1.00)	100 (89.3–100)	100 (89.0–100)
ARGENE	N	0.97 (0.91–1.00)	100 (89.3–100)	96.8 (83.8–99.4)
	RdRp	0.81 (0.67–0.95)	81.3 (64.7–91.1)	100 (89.0–100)
	E	Use for resolution of equivocal samples		
	N + RdRp + (E)	1.00 (0.91–1.00)	100 (89.3–100)	100 (83.8–99.4)
CoronArDx	E	0.94 (0.69 –1.00)	93.8(79.9–98.3)	100 (88.6–100)
	RdRp	0.31 (0.13–0.48)	31.3 (18.0–48.6)	100 (88.6–100)
	E + RdRp	0.94 (0.69–1.00)	93.8 (79.9–98.3)	100 (88.6–100)
Wiener lab	N + RdRp	0.97 (0.91–1.00)	96.9 (84.3 –99.4)	100 (89.0–100)
iAMP COVID-19 Detection Kit	N + ORF1ab	0.93 (0.67–1.00)	92.6 (83.0–100)	100.0 (89.0–100)
NEOKIT	E + N + ORF1aa + ORF1ab	0.75 (0.59–0.91)	75.0 (57.9–86.7)	100.0 (0.89–100)
ELA-CHEMSTRIP	E	0.84 (0.71–0.97)	100 (89.3–100)	83.9 (67.4–92.9)

^1^ SARS-CoV-2 targets: E gene, S gene, N gene, RdRp gene. ORF1ab: 0pen reading frame 1ab. CI95%: 95% confidence interval.

## Data Availability

The raw data used to carry out this work are available upon request.
